# Recent Advances in Lung Ultrasound

**DOI:** 10.3390/diagnostics15212740

**Published:** 2025-10-29

**Authors:** Rosa Cordovilla, Enrique Cases

**Affiliations:** 1 Servicio de Neumología, Hospital Universitario de Salamanca, 37007 Salamanca, Spain; 2 Servicio de Neumología, Hospital Universitario y Politécnico La Fe, 46026 Valencia, Spain; cases_enr@gva.es

Thoracic ultrasonography has evolved in recent years as a diagnostic tool and management aid not only for pleural pathology but also for the chest wall and lungs. This Special Issue exemplifies this development, covering findings in both malignant and benign pathology, the use of ultrasonography in the study of the diaphragm in patients with neuromuscular diseases and in cardiogenic pulmonary congestion, and the use of ultrasound in the operating room to mark the pulmonary nodule that will subsequently be excised.

Möller K. et al.’s work provides an overview of the characteristics of benign pleural pathology, from pleural thickening to benign tumors [1]. Notably, in addition to the features of B-mode images with color Doppler, the characteristics of contrast-enhanced ultrasound (CEUS) are also described. This aspect of the study of transthoracic pathology is increasingly important in thoracic disease diagnosis; thus, the presence of a double arterial supply in the lung from the bronchial and pulmonary arteries makes it an ideal organ for study using contrast-enhanced ultrasound. CEUS can distinguish between vascularized and nonvascularized lesions, as well as necrosis. This aids in the evaluation of mediastinal lesions, which are sometimes large and highly necrotic, and in the lung to differentiate between tumors, abscesses, and pulmonary infarcts, where non-contrast ultrasound has limitations. In another article, the same authors focus on less common malignant pathologies such as pulmonary sarcoma and mesothelioma [2].

In this review, they analyze the appearance of these tumors on B-mode ultrasound (US), color Doppler ultrasound, and CEUS. A key aspect of this work is that they provide guidelines for obtaining histologically evaluable material via CEUS-guided sampling. This is highly relevant when dealing with large masses with abundant necrosis; without CEUS, the yield is lower. Moreover, the diagnostic failure of US-guided biopsy is mainly due to the tumor’s intrinsic heterogeneity, and CEUS helps differentiate viable from nonviable (necrotic or fibrotic) areas, thus improving diagnostic efficacy, especially in larger tumors with necrotic zones [3,4].

The utility of ultrasound (US) for assessing diaphragmatic motion and, in critically ill patients, evaluating weaning from mechanical ventilation has been widely demonstrated [5]. Additionally, in patients with neuromuscular diseases, ultrasound appears to be a good predictor of pulmonary function decline and hypoventilation. The study by Iglesias M et al. aimed to evaluate diaphragmatic ultrasound parameters in ALS, compare them with respiratory function tests, and determine whether they are associated with the need for noninvasive ventilation [6]. A prospective, descriptive, multicenter study observed statistically significant moderate correlations between diaphragmatic excursion and velocity, forced vital capacity, maximal inspiratory pressure, and the SNIP test. The authors conclude that ultrasound can be a useful tool for monitoring patients with ALS.

There is growing interest in applying US during surgery to detect pulmonary nodules. Although other nodule-marking techniques exist, such as computed tomography-guided percutaneous fiducial marker placement [7] or electromagnetic navigation [8], US offers several advantages. In their review, Yasin D. et al. demonstrate that intraoperative lung ultrasound has advantages, such as being a noninvasive, real-time technique with high accuracy, although it is operator-dependent [9]. Image interpretation is not always straightforward; for instance, nodules that are semisolid or have ground-glass opacities have a density similar to that of the adjacent normal parenchyma. Undoubtedly, further studies are needed to improve image interpretation and thus incorporate this promising tool into pulmonary nodule surgery.

In 2008, Lichtenstein D. A. and Mezière G. published a protocol called “Bedside Lung Ultrasound in Emergency” (BLUE), which demonstrated a diagnostic yield of 90.5% for identifying the etiologies of acute respiratory failure [10]. Among the conditions included in the differential diagnosis was heart failure. Pulmonary congestion is a critical factor influencing clinical presentation, therapeutic decisions, and patient outcomes in heart failure. The review by Campora A. et al. [11] shows how US provides a simple, rapid, and accurate method to assess pulmonary congestion, surpassing the diagnostic capabilities of traditional clinical assessment and chest radiography. Furthermore, rapid diagnosis in critically ill patients, where decision-making delay is critical, adds value to US.

The presented Special Issue on pulmonary ultrasonography offers a comprehensive overview of the utility of this tool in clinical practice. US has gained applications as the rich images it provides have been better interpreted. In this respect, we believe this Special Issue of Diagnostics contributes rigor to the existing knowledge on pulmonary US and serves as a guide for its development as well as an incentive for further research.
